# Ocular torsion responses to sinusoidal electrical vestibular stimulation

**DOI:** 10.1016/j.jneumeth.2017.11.012

**Published:** 2018-01-15

**Authors:** Stuart W. Mackenzie, Raymond F. Reynolds

**Affiliations:** School of Sport, Exercise, and Rehabilitation Sciences, University of Birmingham, UK

**Keywords:** VOR, vestibulo-ocular reflex, EVS, electrical vestibular stimulation, Vestibulo-ocular reflex, Electrical vestibular stimulation, Ocular torsion

## Abstract

**Background:**

Eye movements evoked by electrical vestibular stimulation (EVS) offer potential for diagnosing vestibular dysfunction. However, ocular recording techniques are often too invasive or impractical for routine clinical use. Furthermore, the kinematic nature of the EVS signal is not fully understood in terms of movement sensations.

**New method:**

We apply sinusoidal EVS stimuli varying from 0.05 to 20 Hz, and record the eye in darkness using an infrared camera. Eye movement was measured offline using commercially available software to track iris striations. Response gain and phase were calculated separately for eye position, velocity and acceleration across all frequencies, to determine how the brain interprets the EVS signal.

**Results:**

Ocular torsion responses were observed at the same frequency as the stimulus, for all frequencies, while lateral/vertical responses were minimal or absent. Response gain and phase resembled previously reported responses to natural rotation, but only when analysing eye velocity, not position or acceleration.

**Comparison with existing method(s):**

Our method offers a simple, affordable, reliable and non-invasive method for tracking the ocular response to EVS. It is more convenient than scleral coil recordings, or marking the sclera to aid video tracking. It also allows us to assess the torsional VOR at frequencies not possible with natural stimuli.

**Conclusions:**

Ocular torsion responses to EVS can be readily assessed using sinusoidal stimuli combined with an infrared camera. Gain and phase analysis suggests that the central nervous system interprets the stimulus as head roll velocity. Future work will assess the diagnostic potential for patients with vestibular disorders.

## Highlights

• We measured ocular torsion responses to sinusoidal Electrical Vestibular Stimulation.
• Responses were observed at all frequencies from 0.05 to 20 Hz.
• Gain and phase analysis suggest the stimulus is interpreted by the CNS as velocity.
• Our non-invasive method assesses torsional VOR at frequencies impossible with natural stimuli.

## 1 Introduction

Electrical vestibular stimulation (EVS) involves currents applied to the mastoid processes. This modulates activity in the vestibular nerve and, when applied in a binaural bipolar configuration, the brain interprets the signal primarily as head roll motion (Fitzpatrick & Day, 2004). This evokes a compensatory whole-body sway response when standing (Lund and Broberg, 1983, Pastor et al., 1993). It also activates the vestibular-ocular reflex (VOR), predominantly in the torsional plane (Hitzig, 1871, Schneider et al., 2000, Schneider et al., 2002, Watson et al., 1998, Zink et al., 1998, Zink et al., 1997). Although some researchers have suggested that the torsional VOR is largely vestigial in humans (Miller, 1962), ocular recordings during natural vestibular stimulation produce eye/head velocity gain values approaching 1 (Peterka, 1992). This is similar to VOR gain in the yaw and pitch axes, suggesting a functional role for the torsional VOR in maintaining gaze. The EVS-evoked eye movement provides a window into this functional reflex.

Clinical studies have shown that EVS has potential as a vestibular diagnostic (Aw et al., 1996, Aw et al., 1995, Aw et al., 2013, MacDougall et al., 2005, Welgampola et al., 2013). When applied in a monaural configuration (with a reference electrode distant from the ears), diminished EVS-evoked ocular responses have been demonstrated in the affected ears of patients with a variety of vestibular disorders. This includes unilateral and bilateral dysfunction, canal occlusion, vestibular neuritis, canal hypoplasia and vestibular schwannoma (Aw et al., 1996, MacDougall et al., 2005). As described above, the primary ocular response to EVS is torsion. This is more challenging to track than lateral or vertical eye movement, which rely upon pupil translation from video recordings (Karlberg et al., 2000, Quarck et al., 1998). Previous research has often employed invasive techniques such as scleral coils (Severac Cauquil et al., 2003), or directly marking the sclera with surgical pen to facilitate video tracking (Jahn et al., 2003). These techniques are impractical for a routine clinical test of vestibular function. One aim of the current study is to develop a simple, reliable, affordable and non-invasive method for measuring the ocular torsion response to EVS.

In addition to developing a practical method for measuring EVS-evoked ocular torsion, we seek a better understanding of how EVS is interpreted by the brain. As described above, it is well established that the primary EVS sensation is one of head roll motion (Reynolds & Osler, 2012). But whether this motion is position, velocity or acceleration is less well understood. Body orienting responses when stepping on the spot suggest that EVS evokes a sensation of acceleration (St George et al., 2011). On the other hand, motion perception when seated in a rotating chair suggests a signal somewhere between position and velocity, depending upon the stimulus frequency (Peters et al., 2015). Continuous ocular torsional rotation in response to constant-current GVS suggests a velocity signal, rather than a static position signal (Severac Cauquil et al., 2003). Therefore, our secondary aim is to establish the kinematic nature of the EVS signal in healthy subjects. Clarifying this issue in healthy participants will aid interpretation of pathological responses.

So, our first aim is to develop a practical recording technique for EVS-evoked eye movement, and our second is to understand the brain’s interpretation of the EVS stimulus. To address both aims we applied sinusoidal EVS to healthy volunteers using a binaural bipolar electrode configuration. Eye movements were then tracked off-line using commercially available software (Mocha ©;see Osborne and Lakie (2011)). The use of sinusoidal stimuli at multiple frequencies offers two advantages. Firstly, it allows us to validate the tracking technique, since slow-phase eye movement responses should be observed only at the same frequency as the stimulus. Secondly, analysing stimulus-response gain and phase at different frequencies provides insight into how the brain interprets the EVS signal.

## 2 Materials and methods

### 2.1 Participants

9 male participants aged 20–40 years (mean ± SD; 24 ± 6years), with no known neurological or vestibular disorder gave informed written consent to participate. The experiment was approved by the local ethical review committee at the University of Birmingham, and was performed in accordance with the Declaration of Helsinki.

### 2.2 Protocol

**Fig. 1:**
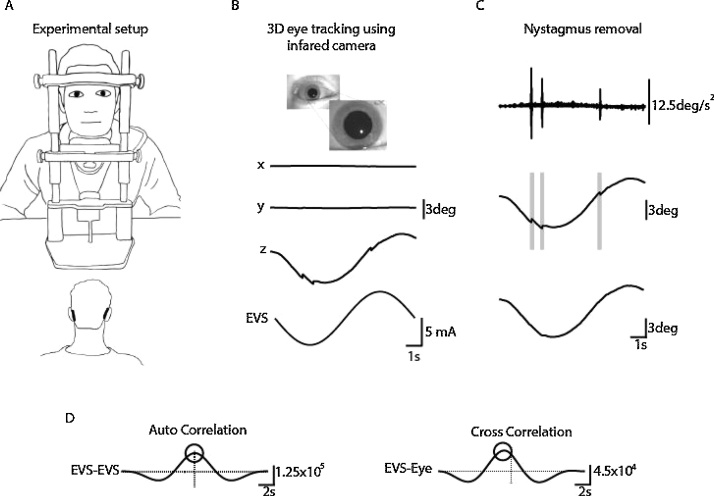
Analysis of EVS-evoked ocular responses. A) Subjects sat in darkness with the head fixed while EVS stimuli of varying frequencies (0.05–20 Hz) were delivered in a binaural bipolar configuration (±5 mA, 10s), B) The eye was recorded using an infrared camera, and movements in all 3 axes were tracked off-line. C) An eye acceleration threshold procedure was used to detect fast phase movements which were then removed using a compensatory inverse nystagmus algorithm. D) Response gain was determined by the ratio of the peak EVS-eye cross correlation to the peak EVS–EVS auto correlation. Phase was determined from the lag of the cross correlation.

Participants were seated with the head restrained (SR Research Ltd. Ontario, Canada) for the duration of each 10 s stimulation period (Fig. 1). Prior to each trial participants were instructed to focus on the lens of an infrared camera and not to blink before being immersed into darkness. An invisible infrared light (940 nm) was used to illuminate the right eye during each trial. No fixation light was provided to ensure that any horizontal and vertical eye movements were not suppressed.

Sinusoidal EVS of varying frequencies (0.05, 0.1, 0.2, 0.5, 1, 2, 4, 6, 8, 10, 20 Hz) were delivered using carbon rubber electrodes (46 × 37 mm) in a bipolar binaural configuration. Two electrodes were coated in conductive gel and secured to the mastoid processes using adhesive tape. Stimuli were delivered from an isolated constant-current stimulator (model 2200; AM Systems, Carlsberg, WA, USA). Positive values of current signify an anode-right configuration. Current amplitude was ±5 mA.

Each stimulus frequency each was repeated three time giving a total of 33 trials. Trial order was randomised and participants were allowed to rest in between trials.

### 2.3 Data acquisition

EVS-evoked horizontal (x), vertical (y) and torsional (z) eye movements were sampled at 50 Hz using an infrared camera (Grasshopper 3, Point Grey research Inc, Richmond, BC, Canada) from the right eye. Eye movements were tracked off-line using commercially available planar tracking software (Mocha Pro V5, Imagineer Systems Ltd. Guildford, UK). Horizontal and vertical movements were tracked by measuring pupil position. Torsional motion was tracked using iris striations. By using sinusoidal stimuli at various fixed frequencies and observing the response at those frequencies, this allowed us to validate the tracking technique (e.g. Fig. 3). Mocha V5 has previously quantified changes in muscle fibre length from ultra sound images which are of similar complexity and quality to our iris recordings (Osborne & Lakie, 2011).

### 2.4 Data analysis

Analysis of the EVS-evoked ocular response is depicted in Fig. 1. For each trial x, y and z components were quantified in degrees of rotation. Position signals were then differentiated twice to give acceleration signals, from which nystagmus’ could be detected. The nystagmus was removed using an inverted nystagmus algorithm. Briefly, the algorithm detects the presence of a nystagmus within the position signal, generates an equal but inverted artificial compensatory nystagmus which is then added to the position signal.

The magnitude of the eye position response was measured as the peak value of the stimulus-response cross-correlation, using the Matlab XCORR function (units in mA deg). To normalise this value with respect to the input stimulus, it was divided by the peak of the stimulus autocorrelation (units in mA^2^). This resulted in a measure of response gain which was independent of trial length (units in deg mA^−1^). The lag of the peak cross correlation was then converted to phase in degrees as follows; Phase (degrees) = 360 x frequency (Hz) x lag(s). In addition to measuring the gain and phase of the eye position response, we performed the same analysis for velocity and acceleration. This was done in order to determine if the EVS signal was closest to position, velocity or acceleration at the various stimulus frequencies. However, instead of differentiating eye position twice to obtain a noisy measure of eye velocity and acceleration, for the phase analysis we integrated the EVS stimulus waveform twice, producing a cleaner waveform.

### 2.5 Statistical analysis

A 1 × 3 repeated-measures ANOVA (SPSS general linear model) was used to compare response gain between the three axes of eye movement (horizontal (x), vertical (y), torsional (z)). All subsequent analysis was restricted to torsion, since this was the only axis in which eye movements were reliably present. A 3 × 11 repeated-measures ANOVA compared gain and phase across measures of response (position, velocity & acceleration) and stimulus frequency (0.05, 0.1, 0.2, 0.5, 1, 2, 4, 6, 8, 10, 20 Hz). Following significant interactions, 1 × 11 repeated-measures ANOVAs were used to investigate effects of frequency separately for position, velocity and acceleration. In all cases, where significant Mauchly’s tests indicated violation of the assumption of equal variances, the GreenHouse-Geisser correction was employed. For all statistical tests, significance was set at p < 0.05. Means and standard deviations are presented in text while means and standard errors of the mean are presented in figures, unless otherwise stated.

## 3 Results

### 3.1 Vestibular-evoked eye movements

**Fig. 2:**
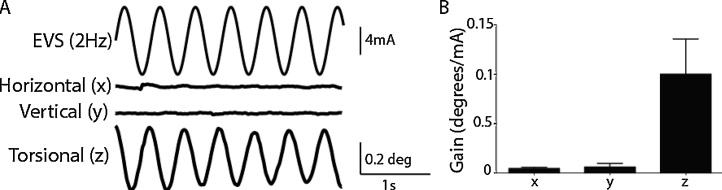
EVS-evoked ocular responses. A) shows horizontal (x), vertical (y) and torsional (z) eye movements for a representative subject evoked by 2 Hz EVS. B) shows mean response gains for each of the three components for this frequency.

Fig. 2A depicts a representative eye movement response from a subject exposed to 2 Hz sinusoidal electrical vestibular stimulation. Horizontal and vertical responses were weak or absent. However, the torsional component was consistently identifiable in all subjects (main effect of axis: F_(2.16)_ = 32.87, p < 0.001). Mean response gain for all subjects is shown in Fig. 2B. All subsequent analysis is restricted to torsional responses.

#### 3.1.1 The ocular torsion response across different stimulus frequencies

**Fig. 3:**
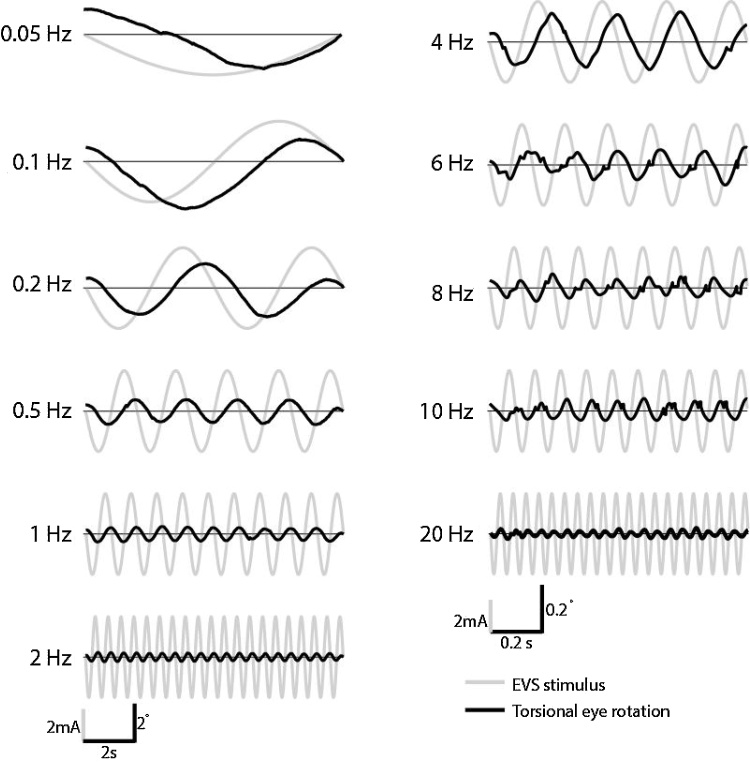
Representative EVS-evoked torsional eye movements across frequencies. A compensatory torsional eye rotation was evoked at all EVS frequencies ranging from 0.05 Hz to 20 Hz. Note the x10 change in eye movement scale between left and right graphs.

The effect of stimulus frequency upon the torsional response is depicted in Fig. 3 for a representative participant. Across all frequencies, an eye movement response can be seen at the same frequency as the stimulus, validating the tracking technique.

### 3.2 Response gain and phase

**Fig. 4:**
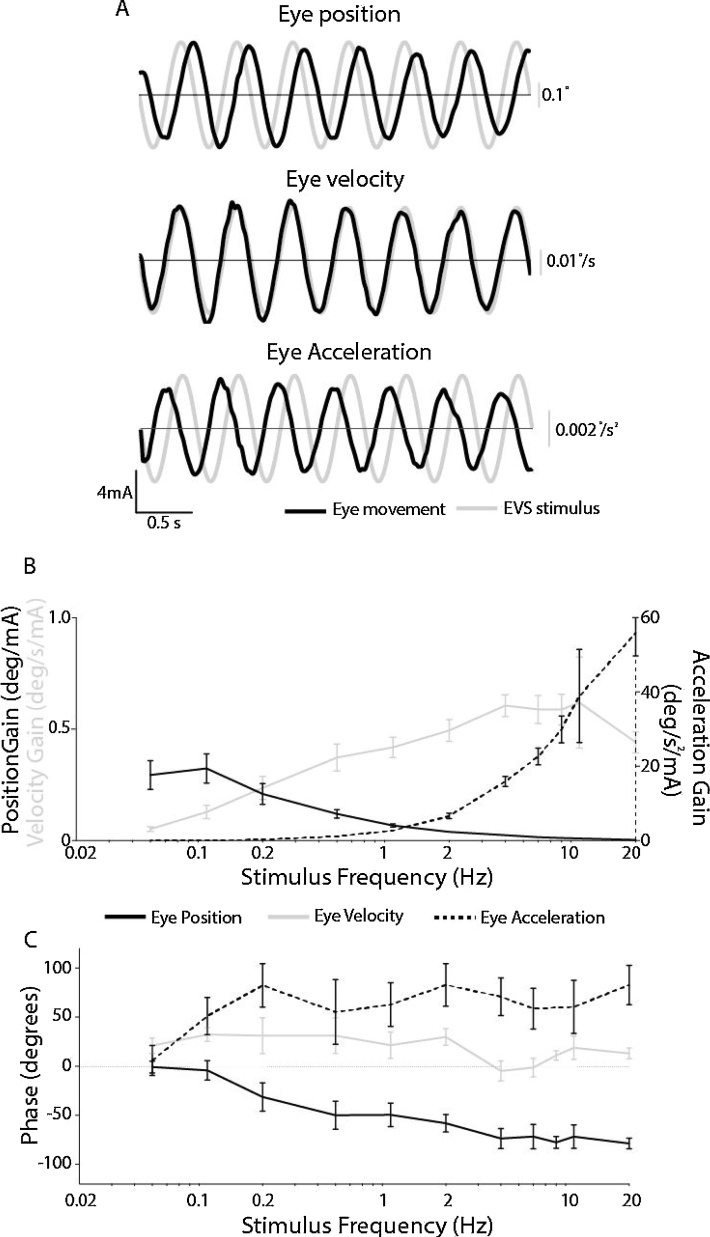
Torsional gain and phase for positon, velocity and acceleration. A) the 2 Hz stimuli and resulting eye movement is shown for a representative subject. B) Mean (±SEM) stimulus-response gain for eye positon, velocity and acceleration. C) Mean (±SEM) stimulus-response phase.

We analysed the gain and phase between the EVS stimulus and the ocular torsion response. This analysis was performed separately for the three response measures of eye position, velocity and acceleration (see Fig. 4A for representative plots). Mean positional gain decreased with frequency (F_(10,80)_ = 17.3, p < 0.001), whereas velocity gain increased (F_(10,80)_ = 8.5,p < 0.001). Acceleration gain also exhibited an increase with stimulus frequency, but with an exponential profile (F_(10.80)_  = 61.3, p < 0.001).

The representative 2 Hz data in Fig. 4A exhibits a phase lag of −107 ° between the EVS stimulus and eye position. This is not apparent in the eye velocity trace, which is almost in phase with the stimulus (+14°). In contrast, eye acceleration exhibits a moderate phase lead of +106 ° with respect to the stimulus. These observations are corroborated by the mean data in Fig. 4C. Positional phase starts around zero degrees for the lowest frequency, increasing to 78 ° at 20 Hz (main effect of frequency: F(10,80) = 10.3, p < 0.001). Eye velocity exhibits a flatter phase plot, with a lead of ∼18 ° and no significant effect of frequency (F_(10,80)_ = 1.2, p = 0.29). Eye acceleration shows a progressively increasing phase lead with frequency, from 5 to 82 ° (F_(10,80)_ = 2.9, p = 0.004).

## 4 Discussion

The commercially available software we used to track the eye has previously been shown to be capable of tracking a variety of biological motion images (Osborne & Lakie, 2011). From our video images, it identified an ocular response at all EVS stimulus frequencies from 0.05 to 20 Hz. In each case, the observed eye movement occurred at precisely the same frequency as the stimulus. This simple observation validates the tracking technique, and confirms that the software did not generate spurious movements. Hence, a relatively cheap off-the-shelf camera in combination with commercially available software was sufficient for reliable measurement of EVS-evoked eye movements in total darkness.

Small vertical eye movements have been reported in response to EVS when using more sensitive (and invasive) techniques such as scleral coils (Severac Cauquil et al., 2003). Along with the much larger torsional component, these disconjugate polarity-dependent movements are consistent with a virtual sensation of roll. They were not reliably detectable in our video recordings, whereas the torsional component was consistently present in all subjects. A small degree of inter-ocular asymmetry in the magnitude of this torsion response has previously been demonstrated (Severac Cauquil et al., 2003). Given that we recorded the right eye only, we could not have seen this. However, this effect was demonstrated with the use of square-wave Galvanic Vestibular Stimulation (GVS), with the left-right magnitude difference observed when comparing cathode-right versus cathode-left stimuli. Such differences are not relevant in our study where the use of sinusoidal stimuli negates any such polarity-dependent effects.

The predominantly torsional nature of the eye movement confirms previous findings, and supports the assertion that EVS induces a sensation of roll motion around a naso-occipital axis, due to activation of canal afferents (Fitzpatrick & Day, 2004). For example, Schneider et al. (2002) showed that the ocular response to a direct-current EVS stimulus was essentially the same as that evoked by natural head rotation in the roll axis. Both stimuli evoked a fixed torsional offset accompanied by nystagmus. Peterka (1992) systematically examined the torsional VOR evoked by chair rotation at frequencies up to 2 Hz, and reported gain values approaching 1. This suggests that the reflex performs a useful function in minimising retinal slip due to head roll, and does not support previous suggestions that it is merely vestigial (Miller, 1962). Hence, by being able to record the EVS-evoked torsional eye movement we gain insight into a functional reflex. Furthermore, it allows us to investigate torsional VOR at frequencies much higher than achievable with a rotating chair.

By analysing response gain and phase as a function of stimulation frequency, we can make inferences about the way in which EVS is interpreted by the brain. When analysed in terms of position, ocular torsion exhibited a steady reduction in gain with frequency. Such low-pass characteristics of EVS-evoked positional responses have previously been demonstrated by Schneider et al. (2000), although they only studied frequencies up to 1.67 Hz. Velocity gain, in contrast, exhibited a steady *increase* with frequency, while acceleration gain showed a much steeper rise. The velocity gain closely resembles the torsional VOR response to natural rotation stimuli, where the ratio of eye velocity to head velocity also exhibits a steady rise with frequency (see Fig. 1 from Peterka 1992). Hence, our gain analysis suggests that EVS current is primarily interpreted as a velocity stimulus. The phase analysis supports this assertion. Eye position exhibited a progressively increasing phase lag with respect to frequency, whereas eye velocity was most in-phase with the stimulus, exhibiting a slight phase lead across all frequencies. Acceleration showed a much larger phase lead, initially increasing with frequency before plateauing. Again, the velocity phase response most strongly resembles the response to natural vestibular stimulation, where eye velocity exhibits a constant small phase lead with respect to rotation velocity, across all frequencies (Fig. 1, Peterka 1992). Hence, both gain and phase are consistent with EVS-evoked changes in vestibular afferent firing rate being interpreted by the brain as a torsional velocity signal.

The stimulation and recording techniques we describe here offer potential for clinical diagnostic use, since it is affordable, non-invasive, comfortable and relatively quick. To assess the function each ear separately would simply require a monaural stimulus, with a reference electrode distant from the ear (Aw et al., 2013, MacDougall et al., 2005)

## Competing interests

No conflicts of interest are declared by the authors.

## Author contribution

This study was performed at the school of Sport, Exercise and Rehabilitation Sciences, University of Birmingham, UK. SWM and RFR contributed to conception and design of the experiments, analysis and interpretation of data; drafting the article; and revising it for important intellectual content. SWM collected and assembled data. Both authors approved the final version of manuscript.

## Funding

This work was supported by the UK Biotechnology and Biological Research Council (BB/P017185/1 & BB/I00579X/1) and the Ménière’s Society. SWM is supported by an MRC-ARUK PhD scholarship(MR/K00414X/1).
